# Poké: Fast and Reliable Neural-Network-Based Identification of Protein Ligandable Sites

**DOI:** 10.1021/acsomega.6c04816

**Published:** 2026-10-23

**Authors:** Enrica Colasurdo, Alberto Boldrini, Luca Terruzzi, Cassiano Langini, Cristina Risueño Fernández, Carmine Varricchio, Erika Primavera, Emilia Cassese, Alexandros Patsilinakos, Giovanni Spagnolli

**Affiliations:** † 695711 Sibylla Biotech S.p.A., Via Lillo del Duca 10, Bresso (Milan) 20091, Italy; ‡ Department of Pharmaceutical Sciences, University of Perugia, Via del Liceo 1, Perugia 06123, Italy; § Department of Pharmacy, University of Naples Federico II, Via Domenico Montesano, 49, Naples 80131, Italy

## Abstract

Accurate identification of ligandable pockets is of key relevance for structure-based drug design; however, their identification remains challenging, particularly for cryptic sites that become accessible only upon conformational changes. Here, we introduce Poké, a high-performance pocket scouting tool that combines energetic mapping with a neural-network-based ligandability score, enabling rapid and reliable binding site detection in both static structures and long-timescale molecular dynamics (MD) trajectories. Poké identifies low-energy regions on protein surfaces through probe-based grid energy calculations, expands and clusters these regions into coherent pocket volumes, and computes physicochemical descriptors that are integrated into a learned ligandability estimator. The scoring model is trained on pockets identified across large data sets using docking-derived binding score distributions, allowing robust ranking of candidate sites. Benchmarking on publicly available data sets demonstrates competitive pocket detection accuracy, improved ranking performance, and substantially enhanced computational efficiency compared to widely used pocket detectors. As a representative application, we apply Poké to mixed-solvent MD simulations of TEM-1 β-lactamase, showing efficient tracking of an experimentally validated cryptic pocket and its dynamic ligandability along the trajectory. Overall, Poké enables scalable, high-throughput ligandability assessment across extensive conformational landscapes.

## Graphical abstract

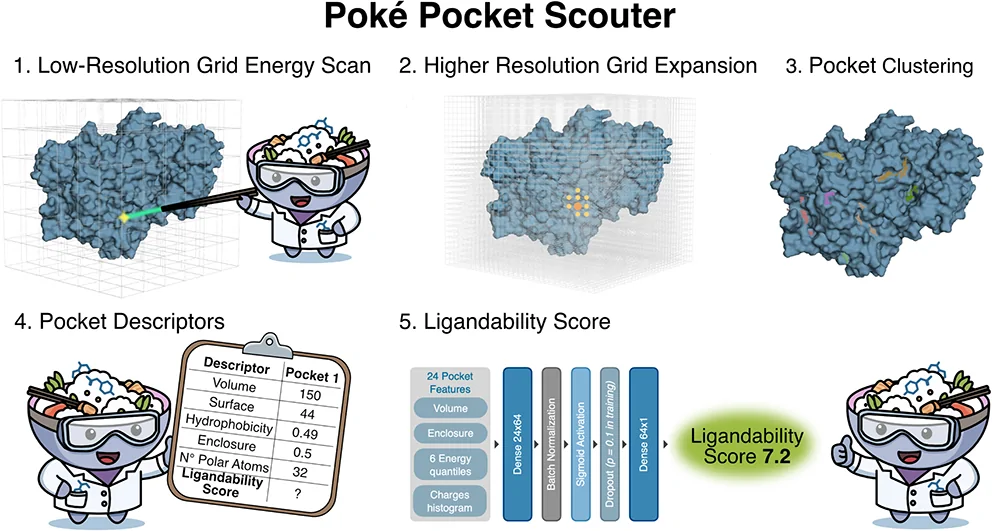

## Introduction

Pocket detection and characterization have become pivotal steps in structure-based drug discovery, enabling the identification and analysis of binding sites on protein surfaces and within protein interiors. These computational approaches facilitate the rational design of small-molecule inhibitors, allosteric modulators, and targeted protein degraders by revealing ligandable regions that can accommodate therapeutic compounds.

Over the past four decades, a series of methodological milestones have shaped the field of computational binding site analysis. The pioneering tools DOCK^1^ and GRID,^2^ developed in the early 1980s, introduced two foundational paradigms that continue to influence modern approaches. DOCK implemented a geometric strategy, leveraging spherical representations to identify potential pockets through shape complementarity, whereas GRID pioneered an energy-based methodology grounded in the molecular interaction field (MIF) approach, mapping interaction hot spots using chemical probes. These two complementary strategies (geometry- and energy-based) continue to define the conceptual landscape of pocket detection to the present day.

Building on DOCK’s geometric foundation, the following two decades saw the emergence of a suite of shape-based algorithms, including POCKET,^3^ SURFNET,^4^ LIGSITE,^5^ PASS,^6^ and CASTp.^7^ These methods define cavities exclusively according to surface topology and volume, independent of physicochemical properties. Their key advantage lies in computational efficiency, making them well-suited for rapid screening across large structural data sets. However, methods based exclusively on geometric criteria frequently identify spurious surface depressions and show limited discrimination between truly ligandable pockets and nonligandable cavities of comparable geometry.^8^ Recognizing the limitations of purely geometric criteria, hybrid strategies such as fpocket^9^ and LIGSITEcsc^10^ combined geometric efficiency with chemical and evolutionary descriptors, substantially improving the discrimination of true ligand-binding sites from false positives. Recent independent benchmarks, which evaluate such methods alongside both purely geometric tools and machine-learning predictors, confirm their broad applicability and continued role as standard references for binding-site identification.^8^ Parallel to these developments, the energy-based lineage of GRID inspired tools such as FTMap,^11^ SiteHound,^12^ FTSite,^13^ and SiteMap,^14^ which employ probe-based mapping strategies to identify biophysically meaningful interaction sites. By locating regions of favorable binding energy rather than mere geometric cavities, these methods offer improved physicochemical relevance and have proved particularly effective for identifying allosteric sites and fragment-binding hot spots.^13,15^ Their main disadvantage is a greater computational cost relative to geometric approaches, which reduces their suitability for large-scale ensemble-based screening.

Advances in structural bioinformatics during the same period also gave rise to template-based methods, including COACH,^16^ FINDSITE,^17^ and MetaPocket,^18^ which transfer binding-site information from structurally homologous complexes. These approaches can achieve high accuracy when close structural analogues are available in existing databases, but their performance degrades substantially for novel or structurally divergent targets, where suitable templates are absent. The 2010s marked a major paradigm shift driven by the emergence of large-scale structural and binding databases, which enabled the application of machine learning to protein pocket detection. Tools such as P2Rank,^19^ DeepSite,^20^ DeepPocket,^21^ and Kalasanty^22^ as well as graph neural network architectures such as PocketMiner^23^ and VN-EGNN^24^ learn spatial and physicochemical features directly from large structural data sets rather than relying on geometric heuristics or structural templates. These approaches have demonstrated improved binding-site identification accuracy compared to geometric and energy-based methods in benchmark evaluations.^19,21^ Nevertheless, their performance remains sensitive to training-data quality and coverage,^8^ and the interpretability of their predictions can be limited, which complicates their application in contexts where a mechanistic understanding of binding is required.

A further class of predictors has emerged most recently, including AF2BIND^25^ and IF-SitePred,^26^ which repurpose the internal representations of large pretrained models of protein sequence and structure rather than learning from binding-site annotations alone. In parallel, all-atom cofolding models such as AlphaFold3,^27^ RoseTTAFold All-Atom,^28^ and Boltz-2 ^29^ place small molecules explicitly, although their accuracy remains closely tied to the similarity of the complex being predicted to those in the training data.^30^ Despite these advances, pocket detection remains intrinsically challenging because of the conformational flexibility of proteins. Binding sites often undergo dynamic rearrangements, with transient or cryptic pockets appearing predominantly in specific conformational states. To capture this complexity, several trajectory-based approaches have extended pocket characterization to the dynamic realm of molecular dynamics (MD) simulations. Tools such as MDpocket,^31^ POVME,^32^ TRAPP,^33^ and PockFlex^34^ operate as postprocessing methods that analyze precomputed MD trajectories, enabling time-resolved characterization of pocket formation and evolution, and offering key insights into the dynamic accessibility of binding sites. However, these approaches entail substantial computational costs, particularly when applied to long trajectories or large protein ensembles, thereby limiting their scalability for high-throughput ligandability assessment.

To overcome this limitation, we developed Poké, a high-performance Python package that integrates CHARMM36m^35,36^ force-field-based energy calculations with machine-learning-derived ligandability scores. By employing an energy-based approach to rapidly identify low-energy binding regions and computing a comprehensive set of pocket descriptors, Poké enables efficient characterization of ligandable sites across extensive MD trajectories at a fraction of the computational cost of existing trajectory-based tools. Its neural network component assigns pocket scores based on learned relationships between pocket features and ligandability, thereby facilitating rapid prioritization of binding sites for drug discovery. Designed for scalability, Poké maintains high accuracy while dramatically reducing computational time, making it suitable for both single-structure analysis and large-scale trajectory screening.

## Methods

To predict ligandable binding sites and assign a quantitative ligandability score, Poké operates through the following steps. First, it builds a 3D grid around the protein and computes a coarse van der Waals energy map (Lennard-Jones potential with CHARMM36m parameters) to identify low-energy “seed” regions, which are then expanded into full-resolution pocket volumes. Identified regions are subsequently clustered by proximity, defining a low-energy “bulk” core and assigning nearby “spot” regions while filtering out negligible cavities. For each pocket, Poké calculates a set of geometric and physicochemical descriptors (e.g., core and accessible volume, enclosure, surface properties, and electrostatic features). Finally, these descriptors are fed into a pretrained neural network that outputs a single ligandability score used to rank and prioritize pockets. The pocket identification algorithm, together with the neural network training procedure, is described in detail in the following paragraphs. Figure 1 provides a schematic overview of the pocket scouting and scoring workflow. To ensure maximum reliability, Poké requires fully protonated structures. An optional flag is provided to automate the hydrogen addition process at runtime.

**Figure 1:**
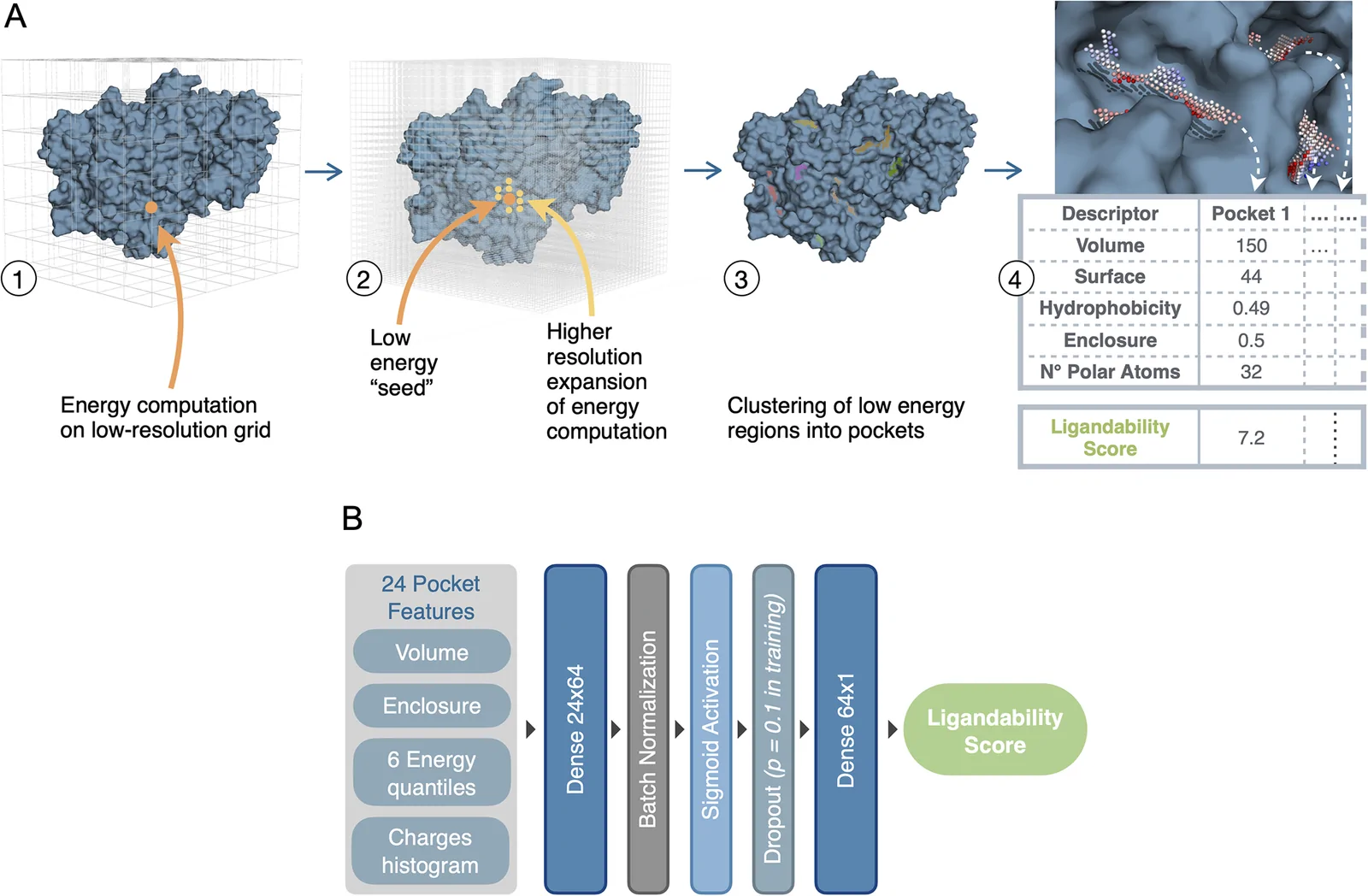
Scouting and scoring algorithm. A) This figure illustrates the pocket identification algorithm used by Poké and the output descriptors for each scouted pocket. From left to right, a proxy of the energy is computed in each point of a 3D grid built around the input protein (1). Each of the points that fall below the threshold of −14.5 kJ/mol is then used as a seed to expand the energy computation on a grid with a 4 times finer resolution than the starting one along each axis (2). Low-energy regions are subsequently clustered into pockets (3), for which Poké computes a set of physicochemical and geometric descriptors and predicts a ligandability score, all of which are reported in the program output (4). The pocket points in (4) are colored according to a modified electrostatic potential (see the Volume Enhancement Procedure section of the Methods). B) The prediction of the ligandability score is conducted with a simple neural network that takes as input selected features of the pocket, i.e., the volume, the enclosure, , the values of 6 specific energy quantiles, and a 16-bin histogram of the charges of the atoms of the pockets.

### Energy Computation on a Lower-Resolution Grid

First, a three-dimensional grid is built around the protein of interest, and a coarse energy map in which energy is calculated every fourth point of the grid for each axis is computed. The current version of the software has a final grid of 0.5 Å spacing; therefore, the low-resolution grid corresponds to a 2 Å spacing. For the identification of pocket regions, energy is computed using a neutral probe; thus, only van der Waals interactions are considered, modeled using the Lennard-Jones potential, as shown in the equation below.

$$V_{\mathrm{LJ}}\left(r\right)=4\epsilon \left[{\left(\frac{\sigma }{r}\right)}^{12}-{\left(\frac{\sigma }{r}\right)}^{6}\right]$$

where r is the distance between two interacting atoms, ε is the depth of the potential well, and σ is the distance at which the potential energy is zero.

In each point of the low-resolution grid, the potential is computed for each protein atom that falls within a 7.5 Å distance from the grid point, a cutoff that was chosen to optimize performance (the list of these atoms is then saved as a neighbor list for further use). While r is simply the distance between the protein atom and the grid point, σ and ε are determined using the Lorentz–Berthelot combination rules. Under these rules, the combined σ is the arithmetic mean of the atom σ and probe σ (σ_probe_ = 3.8 Å) and the combined ε is the geometric mean of their respective well depths, considering a probe well depth (ε_probe_) of 2.5 kJ/mol. Protein atom parameters were sourced from the CHARMM36m force field.

### Volume Enhancement Procedure

The points of the low-resolution grid are expanded into full-resolution pocket regions using a flood fill algorithm. Points with energy below −14.5 kJ/mol that have not been visited yet become the “seed” of a new pocket, which grows until no more points below the same energy threshold can be added. During this process, surface points are identified by counting the number of neighbors with energy above that threshold, along with other pocket properties. For the grid points that fall below the energy threshold, a modified electrostatic potential is computed, summing on all protein atoms that fall within the 7.5 Å cutoff. This potential is described by the relationship shown below, where r is the distance between the grid point and the protein atom, and q is the charge of the atom according to the CHARMM36m force field.

$$V_{E}\propto {\left(\frac{q}{r}\right)}^{6}$$

From this potential, 6 quantiles (specifically quantiles 0.05, 0.1, 0.3, 0.5, 0.6, 0.9) are extracted from the distribution of values for the points belonging to the whole pocket and these are used as features for the multilayer perceptron that predicts the pocket ligandability score. Additionally, these modified potential values are saved in the temperature factor column of the pocket points output pdb file and used to color the points of each pocket that is selected by a Poké run (see Figure 1A, step 4).

### Region Clustering

The identified pocket regions are grouped by proximity into larger pockets. This is performed in a two-step process. In the first step, regions are recursively merged into one cluster if they are within a distance of 1.8 Å, identifying the low-energy core of a potential pocket that we named “bulk”. Each pocket has one and only one bulk region. In the second step, each bulk region is then expanded by nonrecursively considering nearby “spot” regions that fall within 3.6 Å of the bulk region that is being expanded. Specifically, if more than one spot region falls below this threshold, they are assigned to the bulk region in a combinatorial way and all these resulting pocket variations are scored individually. In general, a given spot region can be assigned to multiple bulk regions and thus shared by multiple pockets. Following clustering, any identified pocket with a total volume less than 15 Å^3^ is discarded to filter out insignificant surface indentations. By default, of all the pockets sharing one or more regions, only the one with the highest score is retained. This has the effect of pruning the total number of output pockets, keeping only the best-scoring ones for each ligandable region. This last filter can be turned off when launching Poké to keep all the detected pocket variants. A sensitivity analysis shows that the choice of the main algorithm parameters for the pocket detection is robust (Figure S1). The expansion radius default (3.6 Å) has little effect, whereas the energy-threshold default (−14.5 kJ/mol) sits in a recall-favoring regime. The interaction-cutoff default (7.5 Å) lies at the knee of the accuracy/cost trade-off, since increasing it to 9 Å would increase the scouting time by 61%.

### Computation of Descriptors

For each identified pocket, the following descriptors are computed: core volume (Å^3^) as the total number of grid points belonging to the pocket times the volume of each grid point; accessible volume (Å^3^), provided as an alternative pocket volume measure to include regions capable of accommodating the van der Waals volume of ligand atoms, and it can be computed by counting the number of low-resolution grid points having negative energy within a cutoff distance of the pocket points, times the volume of each grid point (throughout this work we have used 1 Å as the cutoff value); surface (Å^2^) as the number of surface points times the square of the grid spacing; pocket dimensions along the 3 principal axes, computed as the eigenvalues of the covariance matrix of the coordinates of the pocket points; enclosure, computed as exp­(−0.3 × net_norm^2^), where net_norm^2^ is the squared norm of the average of the 5N vectors joining each of the N pocket surface points to each of its five nearest protein atoms, in units of the 0.5 Å grid spacing. The enclosure is a value between 0 and 1 intended as a measure of how accessible the pocket is to the solvent. An enclosure of 1 means that the pocket is completely inaccessible to the solvent; hydrophobicity, computed as the fraction of atoms with absolute charge < 0.3e; number of polar atoms, as the number of atoms belonging to the pocket with an absolute partial charge between 0.3e and 0.7e; number of charged atoms, as the number of atoms belonging to the pocket with an absolute partial charge greater than 0.7e; pocket atoms (optionally provided as output) are selected as the union set of the closest 5 protein atoms, for each surface point; and residues, as the set of residues containing the pocket atoms.

### Ligandability Score Computation

Each valid pocket is subsequently evaluated for its potential to bind a drug-like molecule using a pretrained machine learning model. The ligandability score is predicted by a feed-forward neural network. This model takes the feature vector of a pocket as an input and outputs a single scalar, representing the predicted ligandability. The pocket features are the volume, the enclosure, 6 energy quantiles (quantile 0.05, 0.1, 0.3, 0.5, 0.6, 0.9), and a histogram of the charges, specifically a 16-bin histogram of the pocket atom charges using a bin width of 0.1e. The multilayer perceptron is composed of the following layers: a dense layer, taking 24 features as input and 64 as output, batch normalization taking 64 input values, a sigmoid, a dropout layer for training with a dropout probability of 0.1, and a final dense layer taking as input 64 features and outputting the ligandability score. The choice of a shallow architecture was motivated by the principle of parsimony and the trade-off with computational speed; a sweep over network depth and width (Figure S2) showed that generalization capacity improves only marginally by adding more layers and it plateaus at a layer width of 64 neurons.

We quantified the reliance of the scoring model on each input feature by grouped permutation importance (Figure S3). The analysis shows that predicted ligandability is driven mainly by the pocket’s partial-charge distribution (74.8% of baseline R^2^), with smaller contributions from interaction energy (18.8%), enclosure (14.8%), and volume (12.8%). This ordering may partly reflect group size, as the charge distribution is encoded by 16 bins and the energy by 6 quantiles, whereas enclosure and volume are single features.

### Training Data Set for the Ligandability Score

The model for the prediction of the ligandability score has been trained to predict the 95th percentile of the distribution of CNNaffinity values computed by GNINA, which would result from the docking of a 1555-compound library on a given pocket to be scored. The GNINA CNNaffinity score is a convolutional neural-network-based docking score that predicts the binding affinity of a protein–ligand complex, expressed in p*K*_d_ units. Within Poké, the CNNaffinity is employed as a surrogate ligandability descriptor rather than as an estimate of the affinity of an individual complex: the 95th percentile of its distribution over a drug-like library quantifies the tendency of a pocket to accommodate high-affinity binders, providing a docking-based druggability assessment. The reliability of this descriptor relies on the demonstrated performance of the GNINA CNN scoring function, whose capacity to reproduce experimental binding affinities (p*K*_d_) has been established across structurally diverse protein families, both on the standard CASF-2016 scoring benchmark^37^ and across extensive redocking and cross-docking evaluations,^38,39^ supporting its use as a robust source of relative ligandability signal across heterogeneous targets.

The library was retrieved from ChEMBL (release 33; accessed May 2023) by applying a molecular-weight filter of 250–600 Da and restricting entries to compounds with documented clinical development. The protein structures used for this docking were retrieved from the Binding MOAD^40^ and COACH420 ^19^ data sets. The initial data set developed by the authors of MOAD (LBSp data set)^41^ comprised 2,528 structures, featuring a maximum of 10 holo complexes per protein family; however, we excluded apo structures and, in the process of retrieving ligand coordinates (missing from the original data set), we excluded those containing multiple ligands and those with a multiresidue ligand. To complete the curation process, missing side chains were reconstructed with UCSF Chimera,^42^ alternative conformations were resolved, and hydrogens were added with protonation states assigned via GROMACS,^43^ resulting in a final set of 930 structures, which we refer to as “MOAD930”. The COACH420 and the Astex data sets were also preprocessed, following an identical protocol for structural refinement and protonation. The Astex data set,^44^ used in the benchmarking phase, was not included in the training phase for the purpose of having an independent test set.

In order to build the data set for the training of the ligandability score, Poké was used to identify pockets on each structure of MOAD930 and COACH420, obtaining a total of 6345 pockets. For each identified pocket, a minimal bounding box enclosing the volume points was determined using a greedy minimization procedure in which 20 steps of 0.1 rad rotations along all axes are attempted. Then, all molecules in the compound library were docked in the box using GNINA v1.3 ^39^ with a padding of 3 Å and exhaustiveness of 8, generating 5 poses for each molecule. The target ligandability score for each pocket was then computed as the 95th percentile of all CNNaffinity values produced by GNINA , considering only the highest scoring pose for each molecule. A feature vector was also computed for each pocket, containing the volume, enclosure, quantiles 0.05, 0.1, 0.3, 0.5, 0.6, and 0.9 of the energy, and a 16-bin histogram of the pocket atoms with bin width of 0.1e. A visual summary of the training data set preparation is provided in Figure 2.

**Figure 2:**
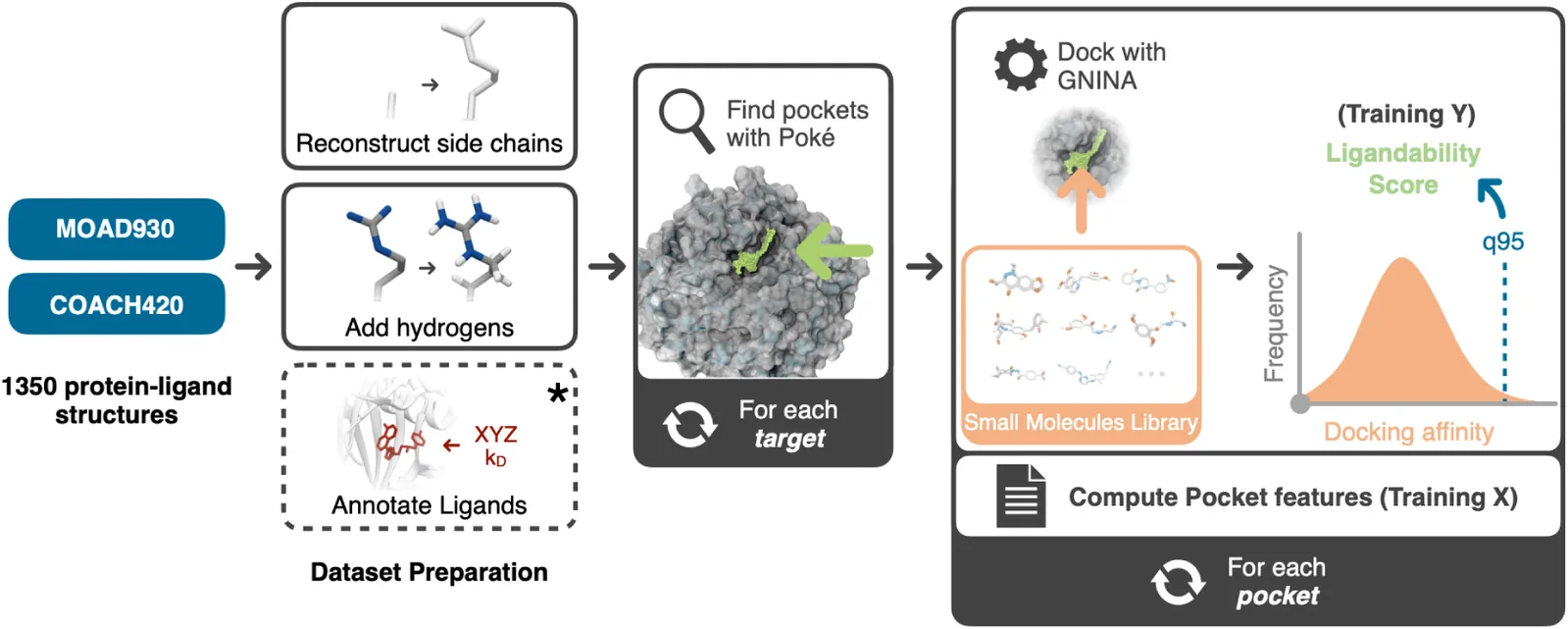
Ligandability score training data set preparation. Protein structures from the MOAD930 and COACH420 data sets were preprocessed by reconstructing missing side chains, adding hydrogens, and annotating the binding affinity of ligands. The combined data set was split into a training, a validation and a held-out test partition. On each target, a pocket detection with Poké was performed. Then, on each scouted pocket, a selected library of 1555 compounds was docked using the GNINA software. The 95th percentile of the resulting CNNaffinity distribution served as the target labels (Y), which the model was trained to correlate with the calculated pocket features (X). The training and validation splits were used for training and model selection, whereas the held-out test set was reserved to quantify the generalization to protein folds not seen during the training. *Ligand annotation was needed only for MOAD930, whose original release lacks ligand coordinates.

The MOAD930 and COACH420 collections, combined into a single nonredundant corpus of 1,350 protein structures, were partitioned into a training and validation split (∼85% of the corpus) and a test held-out set (∼15% of the corpus) under a fold-disjointness constraint. Structures were clustered by fold with Foldseek (v10.941cd33) using its structural workflow (foldseek easy-cluster); two structures were placed in the same cluster when their TM-align alignment exceeded a TM-score of 0.50 over at least 80% of both chains, the established threshold above which two structures are expected to share a fold. Clustering produced 316 fold clusters, ranging in size from singletons to a single dominant cluster of 48 members.

Fold clusters were then assigned to the train or test split, so that every member of a cluster falls on the same side of the partition and the two sides are fold-disjoint by construction. The partitioning was performed based on the constraint that only clusters of at most 10 structures were eligible for the held-out set; larger clusters (including the 48-member cluster) were assigned to the training/validation split. This was implemented to prevent any single fold from dominating the held-out test set, which would couple the performance estimate to the detector’s behavior on one over-represented family. The validation split was obtained by randomly sampling 15% of the training/validation set, without taking fold clusters into account, and it was used for model selection. The held-out set was reserved exclusively for performance assessment and was not used in fitting or model selection. Likewise, the Astex data set was used only as an independent test set. For this reason, we removed from the Astex data set two structures (1KE5 and 1W1P) which were also part of the training split.

Training was performed in 30000 epochs using the Adamax optimization method with a learning rate of 0.0005 and the Mean Squared Error loss function, using the PyTorch Python library. The final model is the one that obtained the highest validation R^2^ during the training. The model’s performance on the train and test splits is shown in Figure S4.

### Benchmark

The benchmark procedure was conducted by assessing the accuracy of Poké in identifying the binding sites of coresolved ligands in three established protein–ligand benchmark data sets, Astex (85 structures), COACH420 (420 structures), and MOAD930 (930 structures), as well as in the fold-disjoint held-out set (199 structures). The Astex data set was preprocessed in the same way as MOAD930 and COACH420, as described in the dedicated section of the Methods.

To systematically assess the performance of Poké, we performed a comparison against other open-source pocket detection software, specifically fpocket v4.0, P2Rank v2.5.1, and Kalasanty. For each protein in the benchmark data sets, binding pockets were predicted independently using the four detection algorithms (Poké, fpocket v4.0, P2Rank v2.5.1, and Kalasanty), with each method generating multiple ranked pocket predictions per protein.

To establish pocket-ligand correspondences, we employed various metrics: DCA, DCC, and volume overlap (for a visual representation, see Figure S5). The Distance from Center to Any (DCA) metric is defined as the minimum Euclidean distance from the geometric center of a predicted pocket to any heavy atom of the cocrystallized ligand. The Distance from Center to Center (DCC) is the Euclidean distance from the geometric center of a predicted pocket to the geometric center of the ligand. A predicted pocket was considered to successfully identify a binding site if DCA (or DCC) <4 Å.

To complement the geometric distance-based metrics, we also performed a volumetric overlap analysis. Ligand and pocket structures were voxelized onto a 3D grid with 1 Å spacing. For each pocket point, all grid points within a radius R were marked as occupied by the pocket, creating a voxelized representation of the binding site. The radius R was set specifically for each method (1.5 Å for Poké, 1.8 Å for fpocket and P2Rank, 1.4 Å for Kalasanty), reflecting the different granularities of pocket representations. These values were chosen after a grid search, and they coincide with the value of radius that corresponds to the maximal area under the curve for that pocket scouter on the COACH420 data set (see Figure S6). On the other hand, all grid points within 1.7 Å from ligand heavy atoms were marked as occupied by the ligand. The intersection volume was computed by identifying voxels occupied by both ligand and pocket representations.

Precision–recall curves were constructed by sorting all predicted pockets across the data set by their scores in descending order. Then, for each point of the curve, precision and recall values are computed for the top scoring N pockets, with N varying in a range that goes from 1 (left side of the graph) to the total number of pockets detected in the whole data set (right side of the graph).

Specifically, for precision–recall curves based on the DCC and DCA metrics, precision was calculated as the fraction of the top N pockets that matched at least one cocrystallized ligand, while recall was calculated as the fraction of cocrystallized ligands that had at least one matching pocket among the top scoring N pockets, whereby the pocket-ligand matching occurs whenever the chosen metric (DCA or DCC) falls below the 4 Å threshold.

Volume-based precision was calculated as the ratio of the intersection volume to the total predicted pocket volume, while volume-based recall was the ratio of the intersection volume to the total ligand volume. This volumetric approach provides a complementary measure of how completely and specifically methods recover the actual binding site volume independent of pocket center positioning.

To assess the impact of any residual similarity-driven bias in the Astex data set, detection accuracy on this data set was evaluated as a function of each target’s structural similarity to the training partition (Figure S7). This was done by assigning the maximum Foldseek TM-score between its ligand-binding chains and any binding chain of the training partition, and evaluating detection performance on cumulative subsets of targets at increasing similarity thresholds. Across all three matching metrics, performance declines only gradually toward the low-similarity end and does not collapse for the targets most dissimilar to training.

The area under the precision–recall curve (AUCPR) using both DCA/DCC and volume overlap served as the primary performance metric, providing a threshold-independent assessment of the ranking quality.

For the analysis of the applicability domain (Figure S8), the fraction of the ligand surface buried by the protein was computed with FreeSASA.^45^ Burial of missed versus detected sites was compared with two-sided Mann–Whitney U tests.

Computational performance was assessed by measuring the total wall-clock time required for each scouter to process the Astex data set, performing all tests on the same computational resources (Intel Xeon Gold 6338 CPU). The resulting runtimes were normalized by the number of entries to yield the mean execution time per entry.

All computational analyses were performed using Python 3.11 with NumPy and SciPy libraries, and visualizations were generated by using Matplotlib. Poké relies on MDAnalysis^100,101^ to read structures and trajectories and to perform atom selections.

### TEM-1 Case Study

TEM-1 β-lactamase was simulated by means of MD in a mixed-solvent environment (30 mM aqueous benzene) to observe the potential opening of an experimentally known cryptic pocket^46^ (PDB ID 1PZO, M182T mutation). The simulation was initiated from the apo X-ray crystal structure^47^ (PDB ID 1LI9, M69V mutation), which has a resolution of 1.52 Å. Structural analysis confirmed that the mutated residues are distal to the cryptic site; thus, no residue replacements were performed to revert the sequence to the wild type. Missing hydrogens were added using GROMACS pdb2gmx and termini were left uncapped. The protein was parametrized with the CHARMM36m force field (version July 2022). The initial structure was then minimized and solvated in a cubic box of 7.6 nm size with TIP3P water and neutralized with a 150 mM concentration of NaCl; additionally, 7 molecules of benzene (for an equivalent concentration of 30 mM) were added at random positions to the simulation box with the GROMACS insert-molecules tool. The system was equilibrated first in the NVT ensemble for 200 ps at 310 K using the velocity-rescale thermostat.^48^ A second step of equilibration was carried out for 200 ps in the NPT ensemble at 310 K and 1 atm, using the Parrinello–Rahman barostat,^49^ with a coupling constant equal to 100 ps. The same settings were kept for the production run, which was carried out for 10 μs with GROMACS 2020, with a saving interval of 10 ps.

An RMSD analysis was conducted for residues 213 to 229, which correspond to the α-helix in the cryptic region, after aligning the backbone of the whole protein.

The residues used for the pocket search with Poké in the cryptic area are residues 221, 225, 229, 230, 231, 248, 250, 251, 261, 263, 279, 282, 283, 286, 287, and 290 (numbering based on crystal1LI9). These were included with the flag-f (filter), specifying that the pockets found should have more than 6 residues in common with this list.

Poké was compared to MDpocket, running both tools on the MD trajectory that was prealigned to the minimized initial apo structure and subsampled at 200 ps. Poké was run with the same filter specified above and the accessible volume was used as the estimator of the pocket volume. MDpocket was run with the recommended two-stage approach: the first run (pocket detection) was used to generate the pocket frequency map. The main volumetric region at isovalue 0.5 in the cryptic site was used to define the area of interest for the second run (pocket characterization). Volume values estimated by Poké and MDpocket were independently normalized to the unit interval [0–1] using a min-max scaling approach. To mitigate the influence of outliers, the 95th percentile was used as the upper bound for scaling rather than the absolute maximum; consequently, all volumes above this threshold were clipped to the normalized value of 1. The Spearman rank correlation coefficient was calculated on the unscaled volumes.

The speed of the two pocket scouters was evaluated on an Intel Xeon Gold 6338 CPU. Poké was run with 32 cores (as a result of the default setting on this system), while MDpocket does not provide a multicore option.

## Results and Discussion

Poké was developed to provide a high-throughput, physics-informed approach to pocket scouting. The algorithm is designed to identify potential ligand binding sites by analyzing the energetic landscape of a protein surface, making it equally effective for static structures and large-scale MD ensembles.

### Pocket Detection Algorithm Overview

The detection algorithm starts by discretizing the space surrounding the protein into a three-dimensional grid. Poké computes a physics-based energetic map, calculating the van der Waals interaction potential between a neutral chemical probe and the protein atoms at each grid point. Low-energy regions, where the probe experiences favorable attraction to the protein surface, serve as the “seeds” for potential pockets.

Once these seeds are identified, they are expanded into full volumetric representations using a flood-fill algorithm. This approach defines the boundaries of the pocket based on a specific energetic threshold, effectively localizing the regions that the heavy-atom centers of a potential small molecule might favorably occupy. Following this, proximal low-energy regions are grouped through a hierarchical clustering process to form the final binding sites. Each site is then evaluated by a multilayer perceptron. This neural network uses the pocket’s physicochemical descriptors, such as its volume, degree of enclosure, and electrostatic profile, to predict a ligandability score (Figure 1). This score provides a measure of the site’s potential to host a high-affinity, drug-like molecule, allowing researchers to prioritize candidate pockets for further study. In particular, the ligandability score is defined as the predicted 95th percentile of the affinity score distribution for a library of compounds docked into such site with GNINA.^38^ The multilayer perceptron that performs this prediction consists of a dense layer, followed by batch normalization, a sigmoid activation, a dropout layer with a dropout probability of 0.1 applied during training, and a final dense layer outputting the ligandability score.

### Training and Benchmark Data Sets

To evaluate the detection performance of Poké and train the ligandability prediction model, we have used established benchmark data sets of protein–ligand cocrystal structures. Specifically, we have considered the Astex diverse set,^44^ a curated collection of 85 high-resolution nonredundant protein structures in complex with drug-like ligands; COACH420, developed by the authors of P2Rank,^19^ which is a widely used data set that includes 420 protein–ligand complexes; and a data set derived from Binding MOAD^40^ built by the authors of MOAD and in which no protein family is over-represented.^41^ Of this data set, we only considered 930 protein–ligand complexes, which we refer to as “MOAD930”. The positions of the cocrystallized ligands of these data sets have been used as reference to benchmark the accuracy of Poké in detecting binding sites. The COACH420 and the MOAD930 data sets were also used to build the training set for the model predicting the ligandability score. Pocket-detection models are particularly susceptible to information leakage: homologous proteins frequently share highly conserved cavities, so a test protein with a close structural relative in the training data yields an optimistically biased estimate of generalization. To guard against this, we partitioned the combined MOAD930 and COACH420 corpus into a training/validation set and a held-out set under a strict fold-disjointness constraint: structures were clustered by fold, and whole clusters were assigned to a single side of the partition, so that no protein in the held-out set shares its fold with any training protein.

The ligandability scoring network was trained only on pockets from the training set, with model selection performed on a validation subset carved from it; the fold-disjoint held-out set and Astex were used exclusively for the final evaluation.

For this specific purpose, all the cocrystallized ligands were removed from the structures and Poké was used to scout for pockets on these proteins prior to docking. The ligandability score data set was then built extracting selected features of the identified pockets (volume, enclosure, 6 energy quantiles, and a 16-bin histogram of pocket atom charges) and constructing the learning target for each pocket by first docking a library of compounds with GNINA on that pocket and then computing the 95th percentile of the distribution of the affinity scores (Figure 2). On proteins of unseen fold, the network reproduces its docking-derived target with R^2^ = 0.76 on the held-out set (R^2^ = 0.87 on the training set; Figure S4). Detection accuracy under the same leakage-controlled protocol, evaluated on both the independent Astex benchmark and the fold-disjoint held-out set, is reported in Figure 3. Figure 4 also reports the performance on the MOAD930 and COACH420 data sets, alongside that on the Astex and the held-out set.

**Figure 3:**
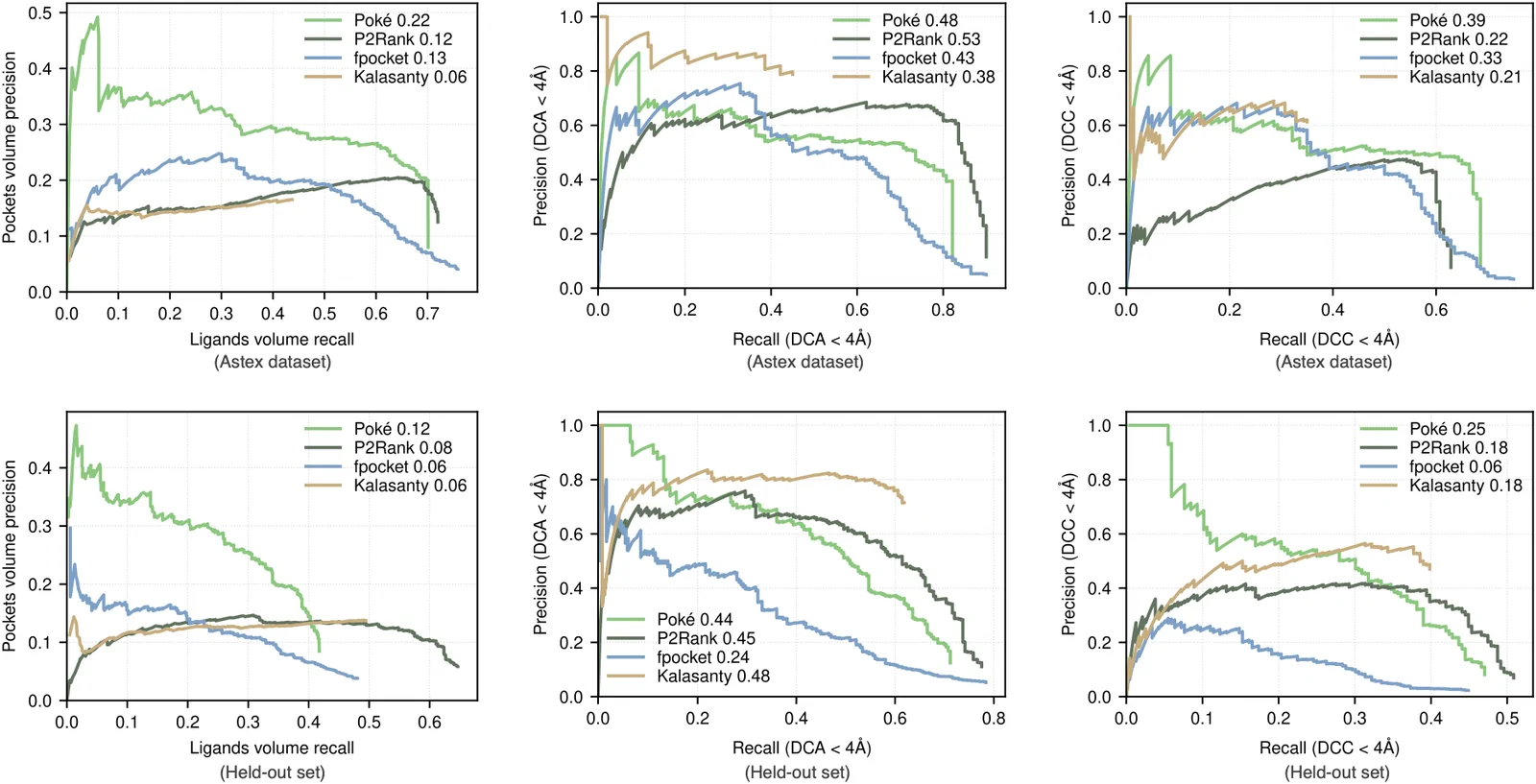
Precision–recall curves. This figure shows precision–recall curves and the corresponding average precisions, computed from Poké, P2Rank, fpocket, and Kalasanty results on the Astex data set and on the held-out set obtained by varying the pocket score threshold. Precision indicates the fraction of pockets correctly identifying cocrystallized ligands. Recall indicates the fraction of correctly identified cocrystallized ligands. Top row: the used data set is Astex. Bottom row: the used data set is the held-out set. First column: a measure of volume overlap between pockets and ligands is used for computing precision and recall. Poké shows the best performance overall, outperforming the other tested scouters, particularly at low recall values. Second column: the DCA measure is used. A ligand is considered identified when the distance between its closest atom to the center of a pocket and such a center is within 4 Å. Third column: the DCC measure is used. A ligand is considered identified when the distance between its center and the center of a pocket is within 4 Å. For DCA and DCC, Poké shows good performance, being among the top scorers in terms of average precision. Notably, Poké is still significantly more precise than the alternatives when only a few top-scoring pockets are considered (low recall region, left of the graphs).

### Accuracy and Speed Benchmark

To evaluate the predictive accuracy and ranking capabilities of Poké, we conducted a comprehensive benchmark against three established pocket detection tools: fpocket (geometry-based), P2Rank (machine-learning-based), and Kalasanty (deep-learning-based).

Pocket identification accuracy was quantified using two standard distance-based metrics: the Distance from Center to Any (DCA) and the Distance from Center to Center (DCC). For DCA, a prediction is considered successful if the geometric center of the predicted pocket lies within a specified threshold of any atom of the cocrystallized ligand. Conversely, DCC measures the Euclidean distance between the geometric center of the predicted pocket and the geometric center of the ligand. For both metrics, we used a threshold of 4 Å, as is commonly used for evaluating the accuracy of pocket detectors.

Beyond these two metrics, we assessed the volumetric fidelity of the predictions, that is, how well the identified pocket points overlap with the actual space occupied by the ligand in the experimental coresolved structure. This metric was quantified through a volumetric overlap analysis comparing the intersection of the predicted pocket and the experimental ligand voxels computed by discretizing the ligand atoms on a grid.

Across all data sets, Poké demonstrated competitive performance, particularly in its high-ranking power. Figure 3 recapitulates the overall accuracy of Poké showing precision–recall curves for the three above-mentioned metrics (DCA, DCC, volume overlap), computed on the Astex data set and on the held-out set. While the overall accuracy for the DCA and DCC is comparable to that of the best-performing pocket scouter (P2Rank or Kalasanty), Poké exhibits improved ranking accuracy, with a remarkably higher precision for top-ranked pockets. Moreover, the performance of Poké stands out compared to the other tested software in the volume-overlap metric. A summary of these results can be found in Figure 4 (top and bottom left). A caveat applies to the volume-overlap comparison. Volumetric overlap metrics are inherently sensitive to how each method represents a pocket: Poké returns a dense, grid-based set of volume points, whereas fpocket describes pockets by alpha spheres and P2Rank by ranked surface points. A voxelized overlap measure therefore aligns more naturally with Poké’s grid-based representation. To limit the resulting bias, the voxelization radius was optimized separately for each tool on COACH420 (Figure S6), so that every method is evaluated at its own peak volumetric performance; the radius selected on COACH420 was then applied unchanged to Astex, MOAD930, and the held-out set. Nonetheless, this per-method optimization cannot fully remove the structural advantage of a grid-based output. Nevertheless, we note that Poké’s dense volumetric representation is a deliberate design to enable high-fidelity tracking of pocket volume along molecular dynamics trajectories, the setting for which this tool excels (see the TEM-1 case study).

Along with higher precision, Poké excels in computational speed. The bottom-right panel of Figure 4 shows the result of the benchmark performed on the Astex data set. Poké is faster than the top-performing scouter when run on individual structures, and its advantage becomes more pronounced when applied to MD trajectories, which are supported as input by Poké and processed taking advantage of multicore parallelization.

**Figure 4:**
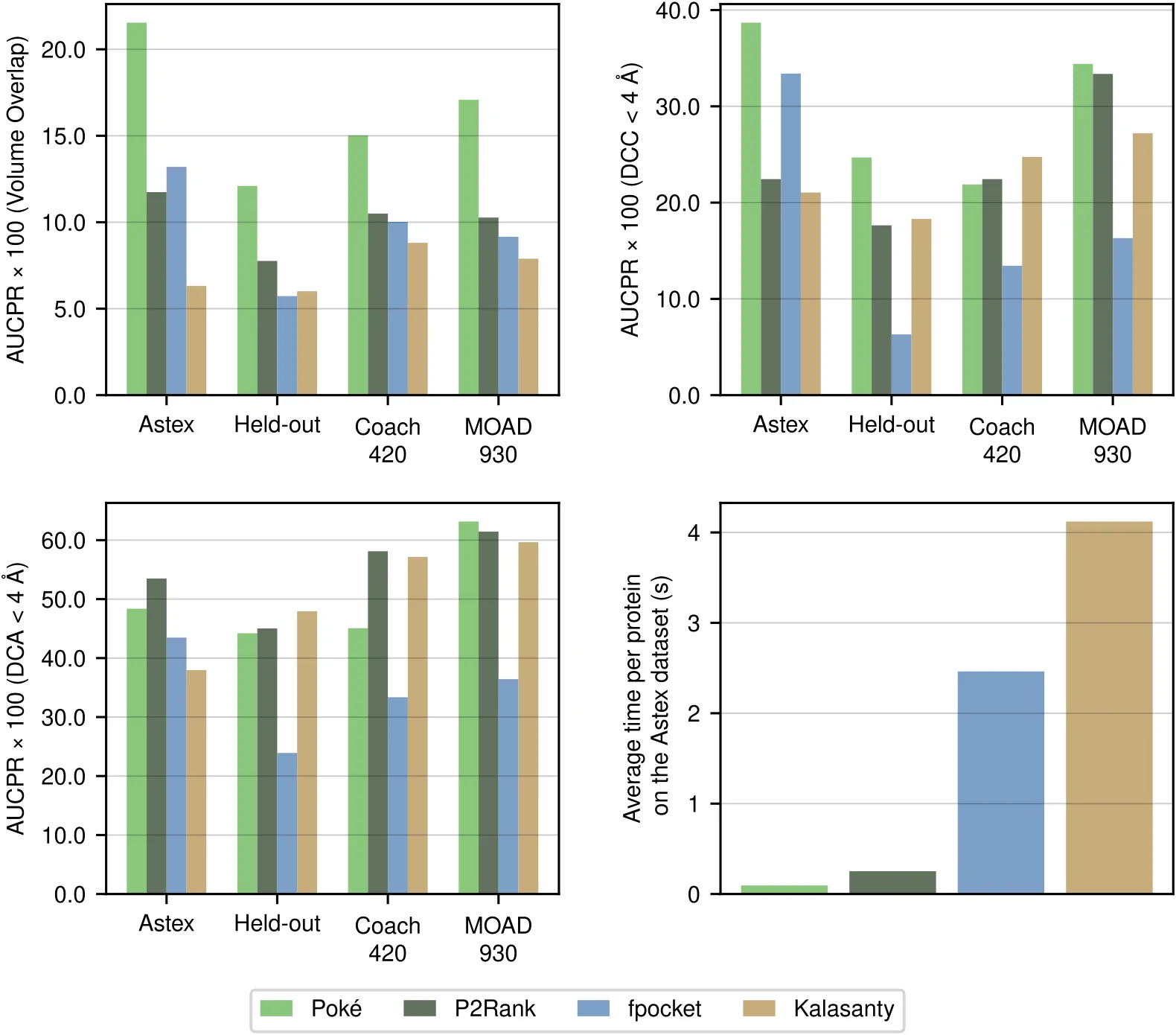
Benchmark of the Poké algorithm. Benchmark comparison for Poké, P2Rank, fpocket, and Kalasanty. The area under the precision–recall curve (AUCPR) is computed with volume-overlap (top left), DCC (top right), and DCA (bottom left) metrics for the Astex, held-out set, MOAD930, and COACH420 data sets. Poké shows good performance, being close to the top performers for the DCA measure and outperforming them on all four data sets in the volume overlap case. The computational speed of the pocket scouters is benchmarked on the Astex data set and the average execution time per structure is reported (bottom right).

Detection is model-independent and generalizes well, with per-receptor DCA recall of 0.82 on the held-out set and 0.87 on the external Astex set; its failures stem mainly from solvent exposure, with highly solvent-exposed pockets over-represented among the missed sites, signaling a potential limitation for low-enclosure cavities (Figure S8). Poké should therefore be applied with confidence to buried, well-enclosed pockets and with caution to shallow or solvent-exposed grooves.

### Case Study

To quantify the computational efficiency of Poké when run on trajectories and further characterize its use in a real-case scenario, we propose a case study on TEM-1 β-lactamase. TEM-1 is a widely studied enzyme that confers resistance to β-lactam antibiotics in Gram-negative bacteria and has long served as a model system for exploring protein structure, dynamics, and ligand recognition. Beyond its well-characterized catalytic site, structural elucidation studies have uncovered the presence of a cryptic allosteric pocket that is not apparent in apo structures but becomes accessible upon ligand binding.^46^ This binding site, which is inaccessible to solvent in the apo state (Figure 5A, left; PDB ID 1LI9), becomes exposed through a marked structural reorganization induced by ligand binding.

**Figure 5:**
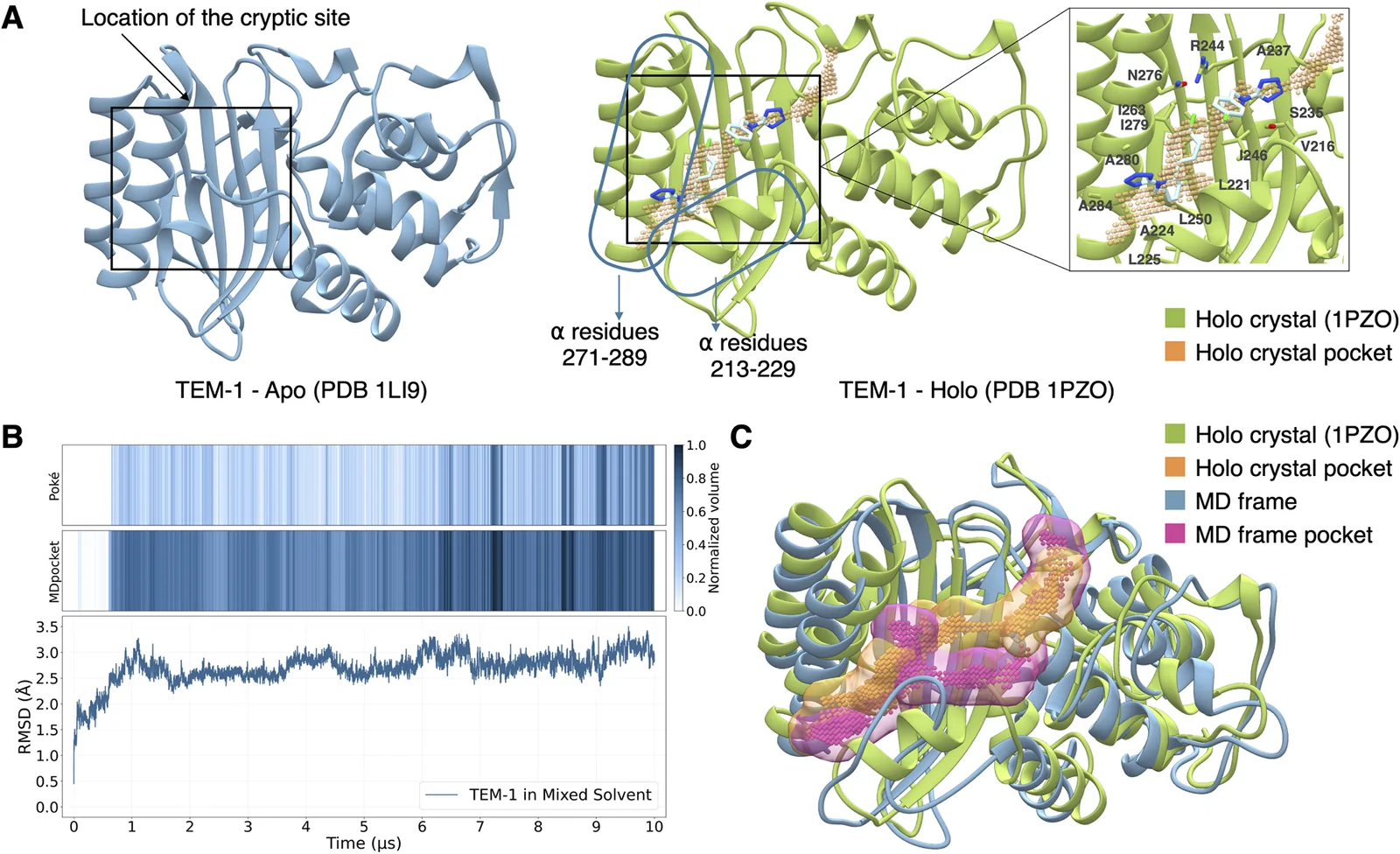
TEM-1 β-lactamase case study. A) TEM-1 presents a cryptic cavity (square) close to the catalytic site, which is not normally accessible, as observed in the apo crystal structure (PDB 1LI9, blue cartoon). However, small molecules can induce the opening of this cavity by displacing the helix corresponding to residues 213–229, as observed in the holo crystal structure of TEM-1 in complex with two allosteric inhibitors (PDB 1PZO, green cartoon and blue sticks, heteroatoms colored by element). Orange dots represent the volume points of the pocket identified by Poké on the holo crystal, which comprises both the cryptic and the catalytic site. Residues delimiting the pocket are highlighted in the inset in sticks. B) Line plot of the RMSD (moving average, 1 ns window) of the α-helix covering the cryptic region (residues 213–229) upon alignment on the protein backbone. The heatmaps show the opening of the cryptic pocket, reported as the normalized volume of the pockets identified by Poké (top) and MDpocket (middle). C) Comparison of an example cryptic pocket (protein in blue cartoon, pocket in magenta surface and dots) found by Poké along the MD trajectory with the pocket of the holo crystal 1PZO (protein in green cartoon, pocket in orange surface and dots).

This transition is driven by a relative displacement of the α-helix spanning residues 213–229 with respect to the α-helix encompassing residues 271–289, ultimately rendering the surface ligand-accessible, where two ligands are cocrystallized in PDB ID 1PZO (Figure 5A, right). In the latter, after reconstructing missing hydrogens with GROMACS, Poké detects a large binding site of 260 Å^3^ (accessible volume), with a Poké ligandability score of 6.6, encompassing both the cryptic site bound by the two ligands and the catalytic site.

Conversely, pocket detection on the 1LI9 apo structure returns only the catalytic site: without a bound ligand, the cryptic site is effectively closed, and no cavity is present.

In general, one viable approach to the detection of such sites could rely on methods that infer binding residues from learned representations of sequence and structure, rather than on the geometry of a cavity, which in principle allows a site to be flagged even where the input structure presents no well-defined pocket.^25,26^ However, achieving complete conformational independence remains challenging. We applied AF2BIND to the same apo structure (1LI9), and its recovery of the allosteric site drops from 0.68 on the holo form to 0.36 on the apo form, with the highest-probability residues concentrated on the catalytic site (Figure S9). A cryptic site that is closed in the available structure may therefore remain invisible to either of the approaches. A well-tested strategy is to characterize the conformational variability of the protein by means of molecular dynamics simulations or equilibrium ensembles generated by deep-learning emulators^50^ and assess whether any open cavities are sampled.

To illustrate the practical usefulness of Poké in analyzing large conformational ensembles, we applied the method to study the dynamic ligandability of the cryptic pocket in TEM-1 β-lactamase using MD simulations under mixed-solvent conditions. To this end, TEM-1 β-lactamase was subjected to a 10 μs cosolvent MD simulation in 30 mM aqueous benzene, starting from the apo crystal structure.

To capture the behavior of the cryptic pocket, Figure 5B reports the RMSD of the α-helix spanning residues 213–229 relative to the protein backbone, together with a heatmap showing the normalized accessible volume of the pockets identified by Poké beneath this helix. This analysis reveals a clear temporal correlation between helix displacement and cryptic pocket formation: during the initial ∼0.7 μs of the simulation, the cryptic site remains inaccessible, as no pockets are detected by Poké in this region. Upon stabilization of the helix RMSD at its plateau, the cryptic pocket becomes accessible and is consistently identified by Poké. To perform a comparison with an established pocket scouter when dealing with MD trajectories, we considered MDpocket. The normalized pocket volume calculated by MDpocket in the same region is also reported in Figure 5B (middle). The volume values estimated by the two methods correlate well (Spearman’s rank correlation coefficient of 0.63).

Notably, 27% of the pockets identified by Poké within the cryptic region display both a favorable ligandability score (greater than 5.9) and an accessible volume exceeding 55 Å^3^, highlighting their potential relevance for drug discovery. Performance-wise, Poké took 3 min and 19 s to analyze the 20×-subsampled trajectory of 50001 frames (speed of ∼251 frames/s), whereas MDpocket took 13 h 40 min and 10 s (∼1 frame/s) on the same computational resources. Finally, Figure 5C shows a representative pocket conformation extracted from the MD simulation that includes both the cryptic and the catalytic sites. This pocket displays a close spatial overlap with the experimentally determined poses of the two allosteric inhibitors in the crystallographic holo structure, supporting the effectiveness of Poké in capturing biologically relevant cryptic sites.

## Conclusion

In this work, we introduced Poké, a high-performance, physics-informed method for pocket scouting that combines energetic mapping with a neural-network-based ligandability estimation to enable rapid and scalable binding site identification. Benchmarking on publicly available data sets shows that Poké achieves competitive pocket detection accuracy, with particularly strong ranking performance for volumetric overlap compared to widely used approaches. Importantly, Poké delivers a substantial computational advantage, which becomes especially pronounced when applied to the analysis of large conformational ensembles.

Using TEM-1 β-lactamase as a representative case study, we showed that Poké can efficiently track the appearance of a cryptic pocket along mixed-solvent MD trajectories, revealing a clear relationship between local structural rearrangements and pocket accessibility. In general, Poké can be useful in characterizing the dynamic changes in volume and ligandability of binding sites across a conformational ensemble.

The failures of Poké concentrate on shallow, solvent-exposed regions, where little enclosed volume exists to be measured. On the contrary, recent deep-learning predictors can detect a binding site even in the absence of a well-defined cavity. The two approaches could be used to complement each other, and a natural combined workflow is to nominate candidate sites with a residue-level predictor, including shallow ones, and to characterize them across a conformational ensemble with Poké. This makes per-frame analysis practical thanks to the throughput of Poké: milliseconds per frame on CPU, against tens of seconds of GPU time per structure for an AlphaFold2 forward pass.^25^

Taken together, these results establish Poké as a practical tool for high-throughput pocket and ligandability analysis, supporting both static structures and MD-driven exploration of transient binding sites, with direct relevance for structure-based drug discovery.

## Supplementary Material



## Data Availability

The software and data sets generated during this study are archived in Zenodo. The Poké source code and a simple user guide are available at 10.5281/zenodo.19634786. The code is also publicly released on GitHub at https://github.com/SibyllaBiotech/poke and on PyPI. The complete user guide is accessible from the repository GitHub page. The associated data sets, including the MD trajectories for the TEM-1 β-lactamase case study, are available at 10.5281/zenodo.19635030, alongside the MOAD930, COACH420, and Astex data sets, and the library of compounds used for building the ligandability training data set.

## Notes

Sensitivity analysis of pocket detection (Figure S1); model capacity analysis (Figure S2); network input features importance analysis (Figure S3); correlation of network-predicted and docking affinities (Figure S4); schematic illustrating the pocket identification accuracy measures (Figure S5); grid search to determine the radius for the volumetric overlap accuracy measure (Figure S6); pocket-detection accuracy as a function of structural similarity to the training partition (Figure S7); analysis of the solvent exposure of missed and detected sites (Figure S8); and comparison of Poké and AF2BIND predictions on the apo and holo forms of TEM-1 β-lactamase (Figure S9) (PDF)
