# Supplementary material for: Poké: Fast and Reliable Neural-Network-Based Identification of Protein Ligandable Sites
Source: ACS Omega. 2026 Oct 23;11(43):66283–94. doi: 10.1021/acsomega.6c04816 (PMC13736924; doi:10.1021/acsomega.6c04816)
Supplement: Supplementary file 1 [file ao6c04816_si_001.pdf]

Poké: Fast and Reliable Neural-Network-Based Identification of Protein Ligandable Sites

Enrica Colasurdo‡[1], Alberto Boldrini‡[1], Luca Terruzzi‡[1], Cassiano Langini[1], Cristina Risueño Fernández[1], Carmine Varricchio[1], Erika Primavera[1,2], Emilia Cassese[1,3], Alexandros Patsilinakos[1], and Giovanni Spagnolli[1*]*

‡These authors contributed equally.

**Correspondence should be sent to:*

enrica.colasurdo@sibyllabiotech.it, giovanni.spagnolli@sibyllabiotech.it

AUTHOR ADDRESS

[1] Sibylla Biotech S.p.A., Via Lillo del Duca 10, 20091, Bresso (Milan), Italy.

[2] Department of Pharmaceutical Sciences, University of Perugia, Via del Liceo, 1, 06123 Perugia, Italy.

[3] Department of Pharmacy, University of Naples Federico II, Via Domenico Montesano, 49, 80131, Naples, Italy.

SUPPORTING INFORMATION

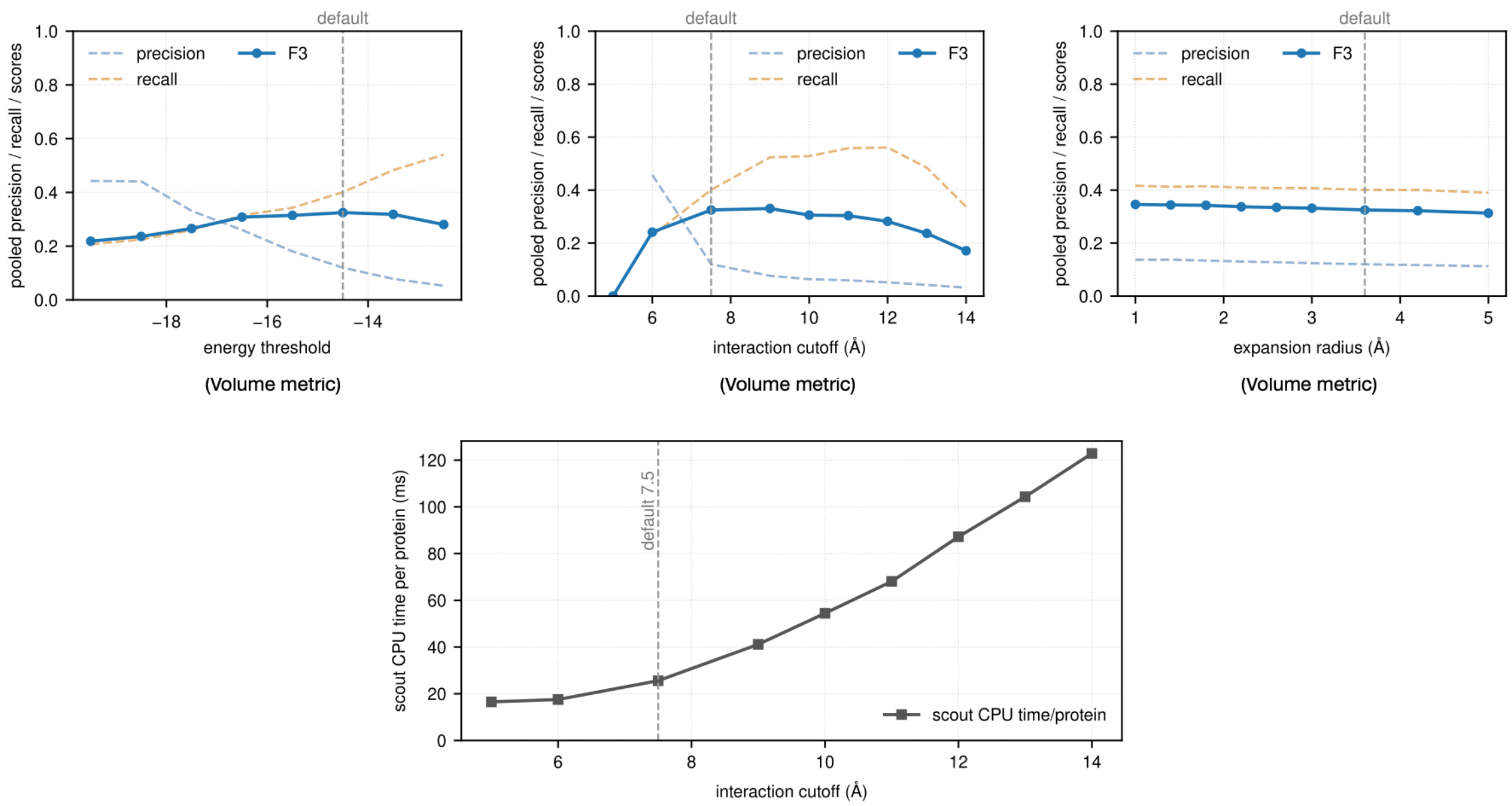

Figure S1. Local sensitivity of pocket detection to the geometric scouting parameters. One-factor-at-a-time sensitivity analysis of the parameters governing Poké's geometric scouting stage, performed independently of the learned scorer. To produce this plot we randomly extracted 100 structures from the COACH420 dataset. Proteins were scouted for each setting variation and returned pockets were evaluated against the experimental ligand sites, without using any score-based ranking or thresholding. Indeed, no model was retrained and these graphs only pertain to the detection capacity of the software. Each parameter was varied over an interval comprising its deployed default (gray dashed vertical line) while the other three parameters were held at their defaults. Detection accuracy was scored against cocrystallized ligands on volume overlap for the three graphs on the top row, with precision and recall pooled (micro-averaged) over the sample. From these we report the F3 score, which up-weights recall

ninefold, with respect to precision. **Top row:** Precision and recall values are computed using the volume-overlap metric (intersection of pocket and ligand voxels on a common 1 Å grid). Columns correspond, left to right, to the energy threshold (default –14.5 kJ/mol), the interaction cutoff (default 7.5 Å), and the expansion radius (default 3.6 Å). These plots show that the deployed defaults are generally positioned to favor recall, consistent for a detector intended to emit candidates generously and delegate precision to the downstream scoring model. **Bottom:** Per-receptor scouting computational cost (median CPU time per protein over 60 receptors, best of five timings) as a function of the interaction cutoff, evaluated on the same set of proteins. Because constructing the energy map scales with the cube of the cutoff, runtime rises steeply beyond the default while accuracy plateaus, placing the 7.5 Å default near the knee of the accuracy/cost trade-off.

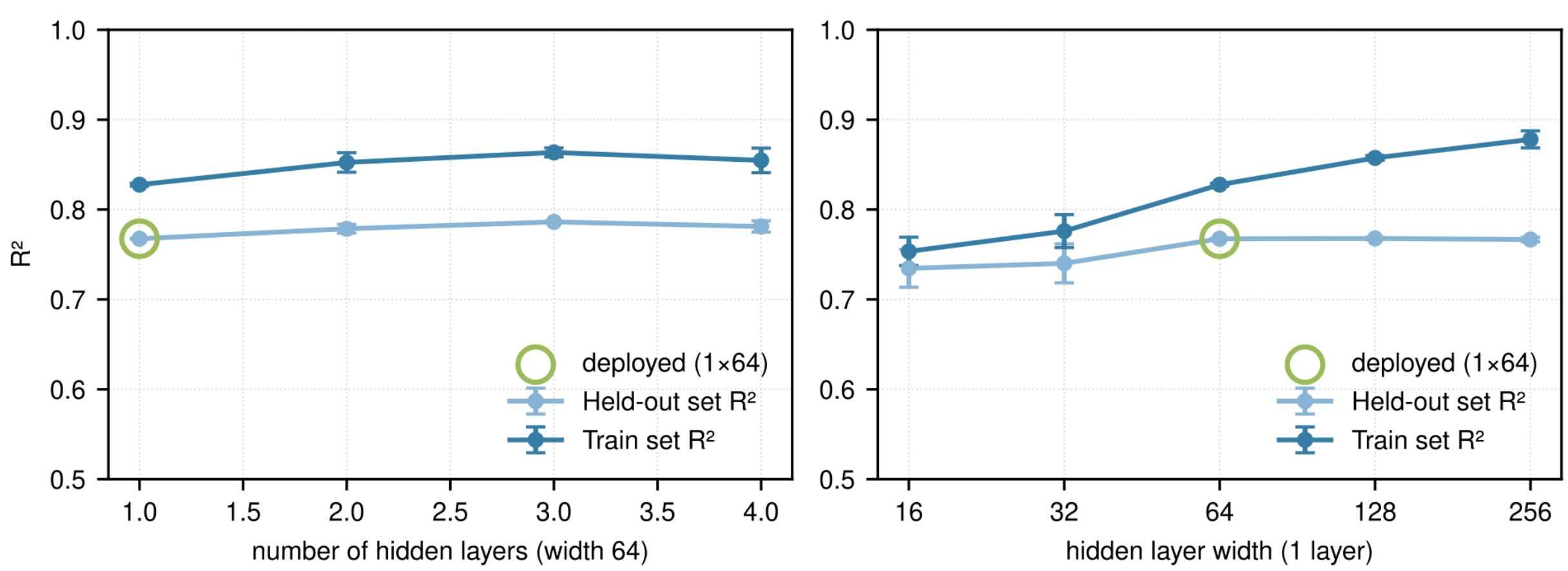

Figure S2. Model capacity analysis. Network accuracy (R^2) as a function of capacity, for a depth sweep (left; 1–4 hidden layers at fixed width 64) and a width sweep (right; a single hidden layer of width 16–256). The deployed architecture parameter, a single 64-unit hidden layer

(1,729 parameters), is circled in green. Models were trained on the training partition (5,303 pockets) to predict the q95 docking-affinity target and evaluated on the fold-disjoint held-out set (1,042 pockets); points and error bars denote the mean ± s.d. across three random seeds. Added capacity improves the fit to the training data (dark curve, rising to $R^2 = 0.88$) but leaves generalization to unseen folds essentially unchanged (light curve): depth contributes at most ~0.02 and plateaus by three layers, while held-out accuracy saturates at width 64, with narrower networks underfitting and wider ones overfitting. Additional parameters therefore buy training fit, not generalization, justifying the deployed network. For this analysis the held-out set is used for both checkpoint selection and reporting, so the held-out values are the best achievable per architecture; this is intentionally conservative, as the absence of any capacity-driven gain holds even under this optimistic estimate. The deployed model is selected on a separate validation set drawn from the training partition (Methods).

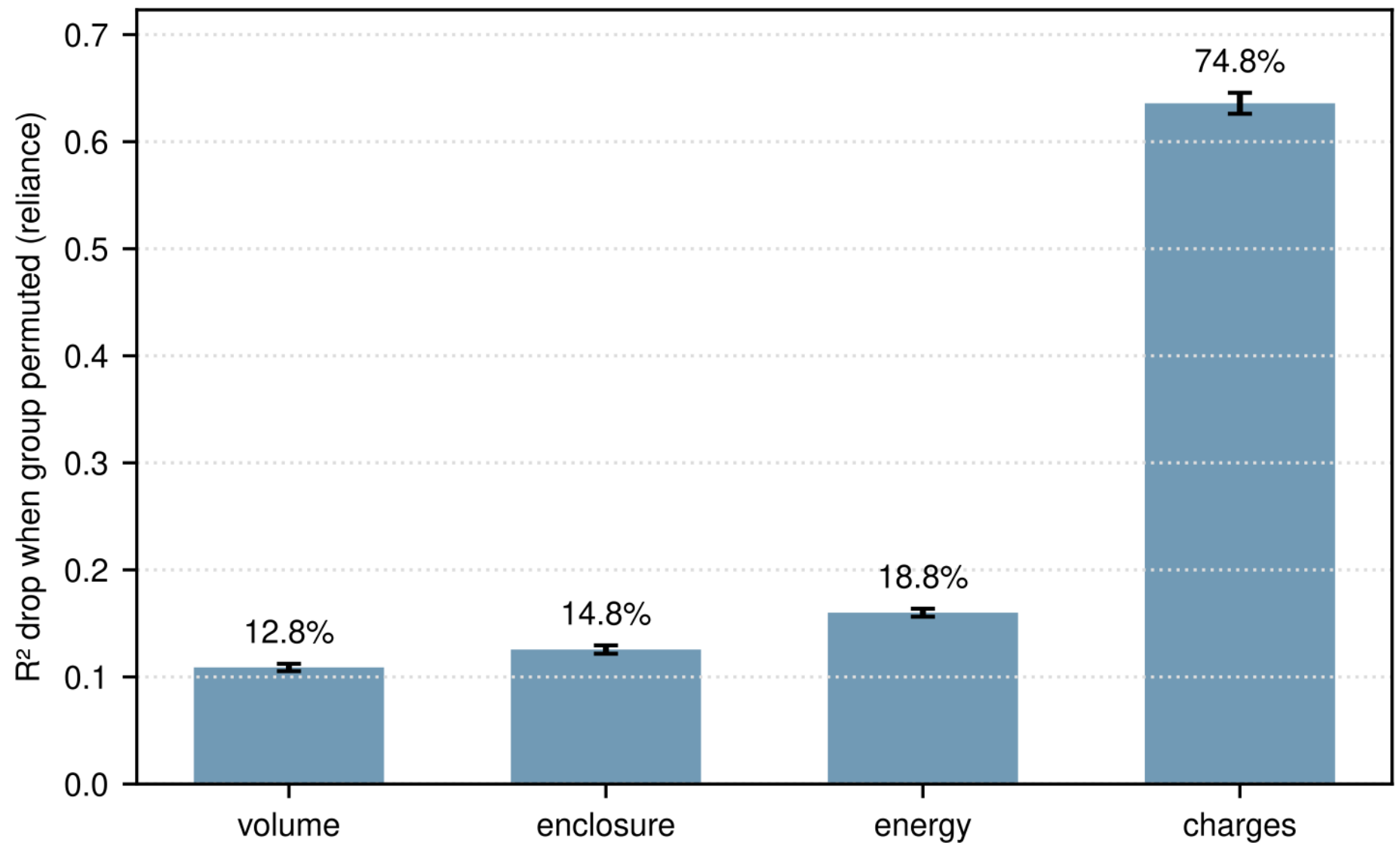

Figure S3. Grouped permutation importance of the ligandability scoring network. Reliance of the deployed scoring network on each interpretable class of pocket descriptor, measured by grouped permutation importance on the full pocket collection (n = 6,345; baseline $R^2 = 0.85$). The 24 input features are partitioned into the four categories: volume (1 feature), enclosure (1), energy (6 quantiles of the per-atom interaction energy), and charges (16-bin histogram of pocket-atom partial charges). For each group, all of its feature columns are jointly permuted across pockets under a single shared row permutation (which destroys the group's association with the target while preserving the internal joint distribution of its columns) and the resulting loss of fit, $\Delta R^2 = R^2_0 - R^2(\text{permuted})$, is taken as the group's importance: the larger the drop, the more the model relies on that group. Bars show the mean ΔR^2 over 50 permutations (seed-fixed) and error bars the standard deviation; the percentage above each bar is that group's importance as a share of baseline performance ($100 \cdot \Delta R^2/R^2_0$). The analysis is performed on the fixed deployed model in inference mode, without retraining, so it quantifies the model's

dependence on each descriptor class rather than the marginal value of refitting without it. Because each group is permuted independently with the others left intact, the four values are not additive and need not sum to 100%. The model relies most heavily on the charge-histogram features (74.8% of baseline R^2), followed by energy (18.8%), enclosure (14.8%), and volume (12.8%).

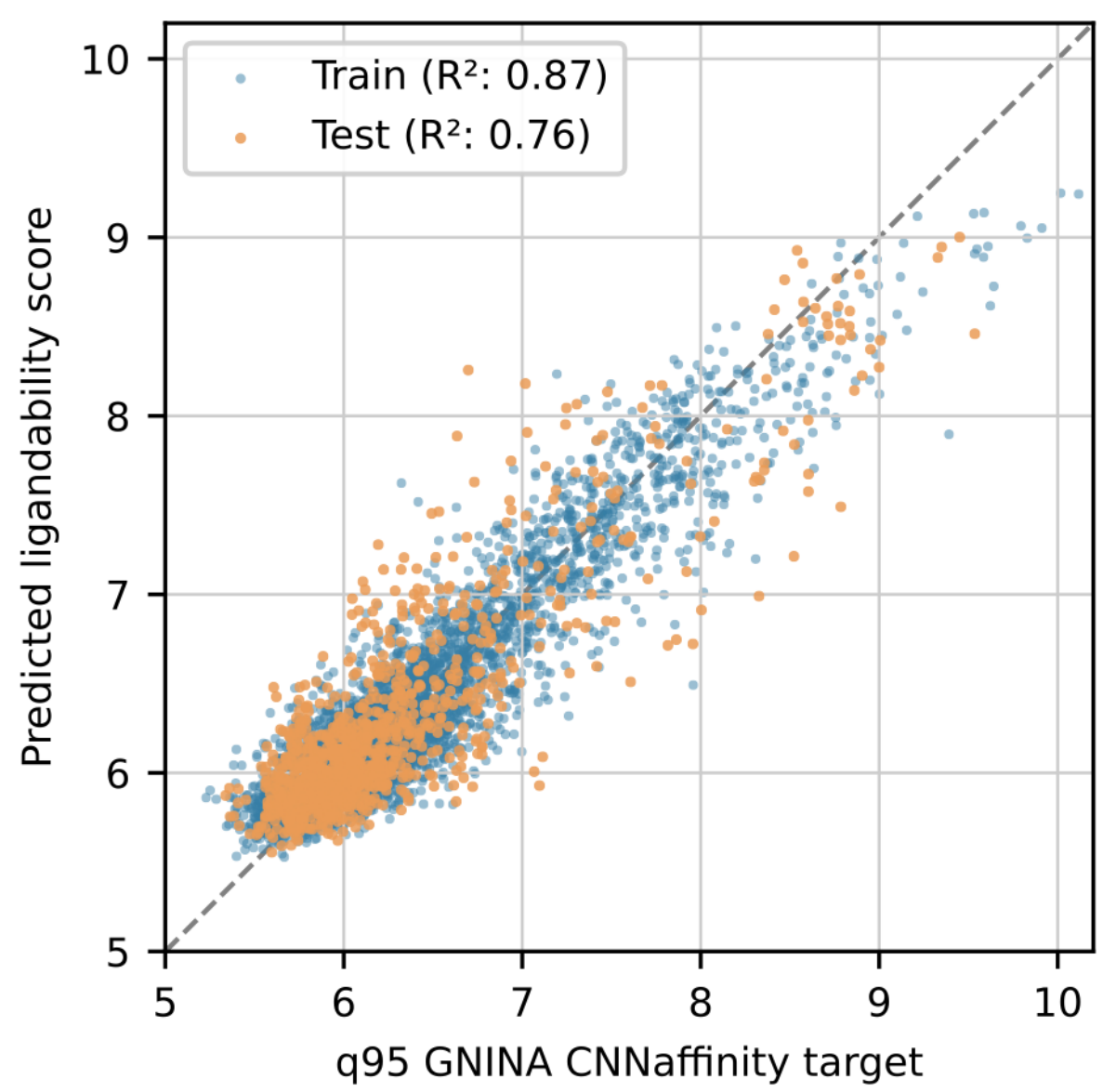

Figure S4. Correlation of predicted affinity vs training/test target values. Comparison of the predicted affinity score by Poké (ligandability score) with the corresponding learning target value: the 95th percentile of the per-compound maximum GNINA CNNaffinity obtained by docking the compound library into each pocket. The train partition consists of 5303 samples (blue points). The test set consists of 1042 samples (orange points) derived from the union of MOAD930 and COACH420 and constituting a fold-disjoint held-out set which is structurally

orthogonal to the train set. Model selection was performed on a separate validation subset of the training partition. The network reproduces the target with $R^2 = 0.87$ on the training partition and $R^2 = 0.76$ on the held-out set, the latter quantifying generalization to proteins sharing no fold cluster with the training data.

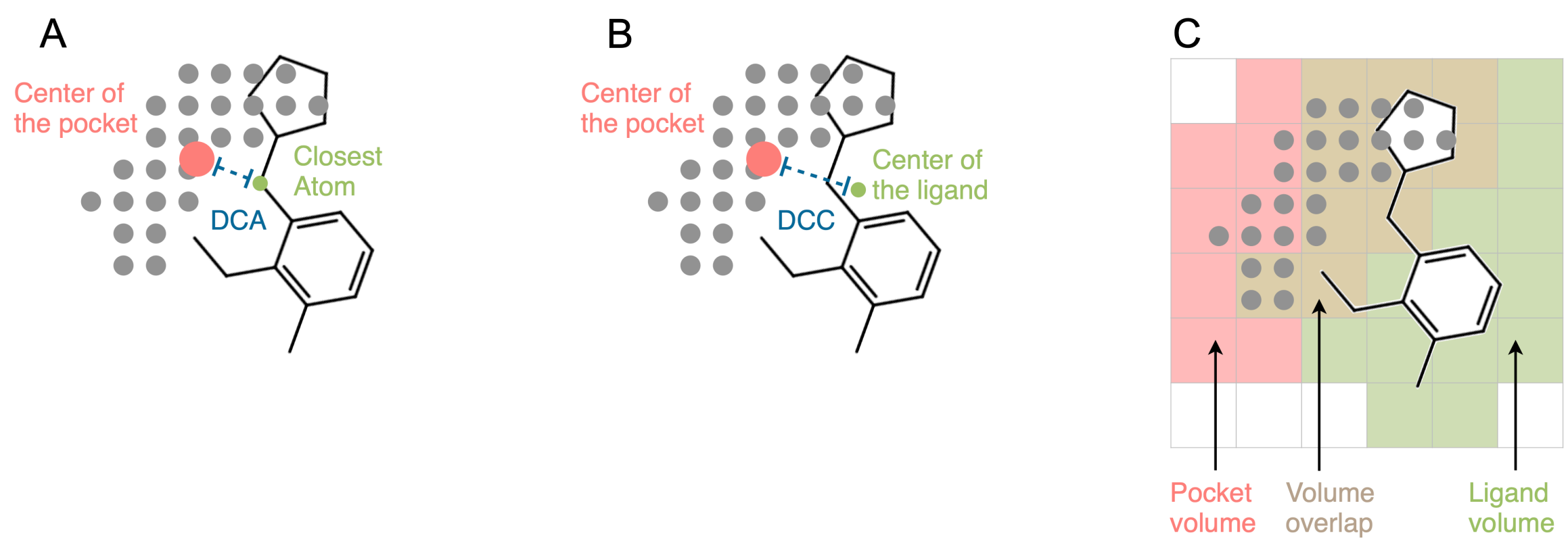

Figure S5. Representation of pocket identification accuracy measures. A) The DCA measures the Euclidean distance between the geometric center of the predicted pocket and the closest ligand heavy atom. B) The DCC measures the Euclidean distance between the geometric center of the predicted pocket and the geometric center of the ligand. C) The volume overlap is a measure of the intersection between the volume of the predicted pocket and the volume of the ligand. To this end, the space is discretized into 3D voxels with 1 Å spacing; all voxels within a radius of 1.7 Å from any ligand atom constitute the ligand volume (light green). All voxels within a given radius R from the predicted pocket points constitute the pocket volume (coral);

R is set specifically for each scouter (1.5 Å for Poké, 1.8 Å for fpocket and P2Rank, 1.4 Å for Kalasanty). Voxels belonging to both the ligand and the pocket contribute to the volume overlap (sand color).

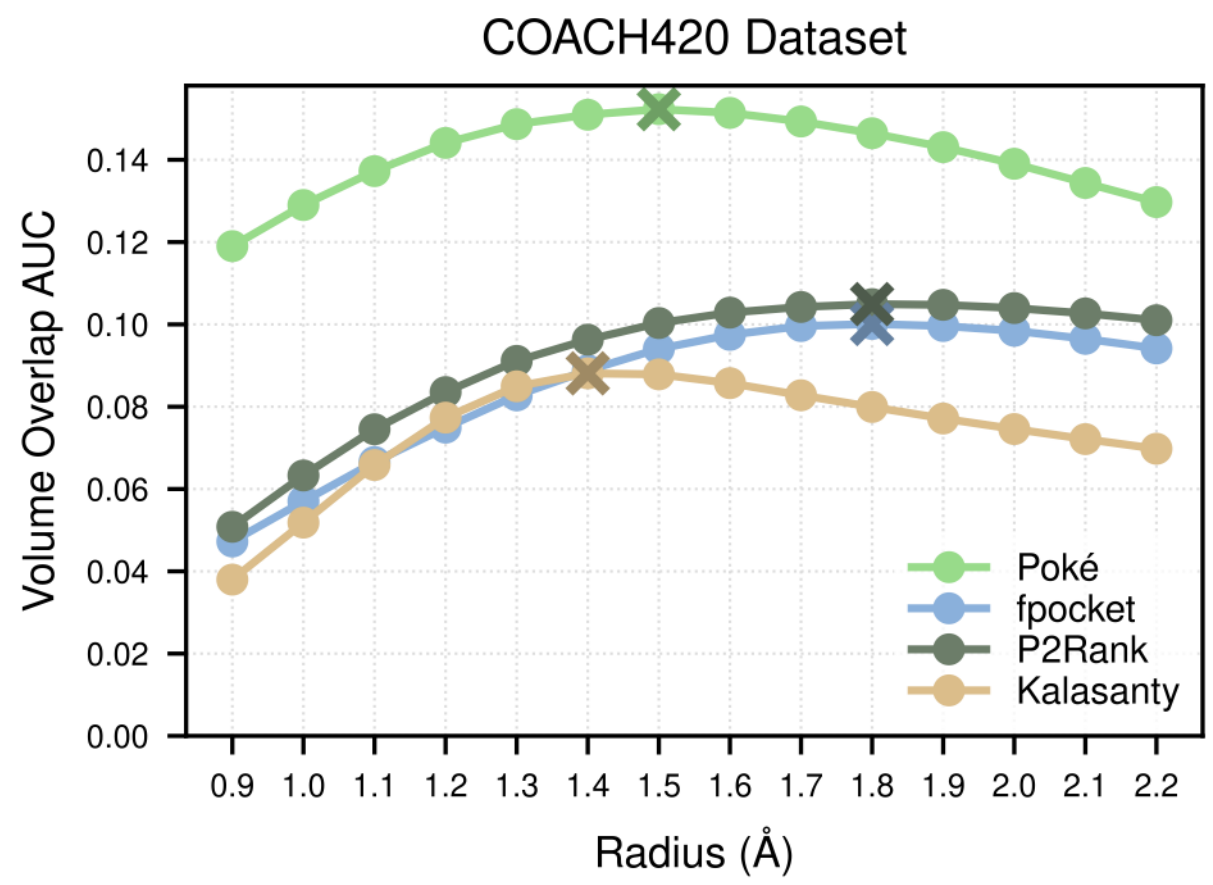

Figure S6. Radius scan for the evaluation of volume overlap precision–recall curves. Grid voxels are assigned to the pocket if they are within a given radius R from any of the predicted pocket points. The value of R is set specifically for each scouter to compare their different pocket representations consistently. The plot shows the variation of the area under the volume overlap precision–recall curve (AUC) as a function of the radius, calculated on the COACH420 dataset. For each scouter, the radius providing the maximum AUC (cross symbol, 1.5 Å for Poké, 1.8 Å for fpocket and P2Rank, 1.4 Å for Kalasanty) was chosen for the analyses reported in the main text.

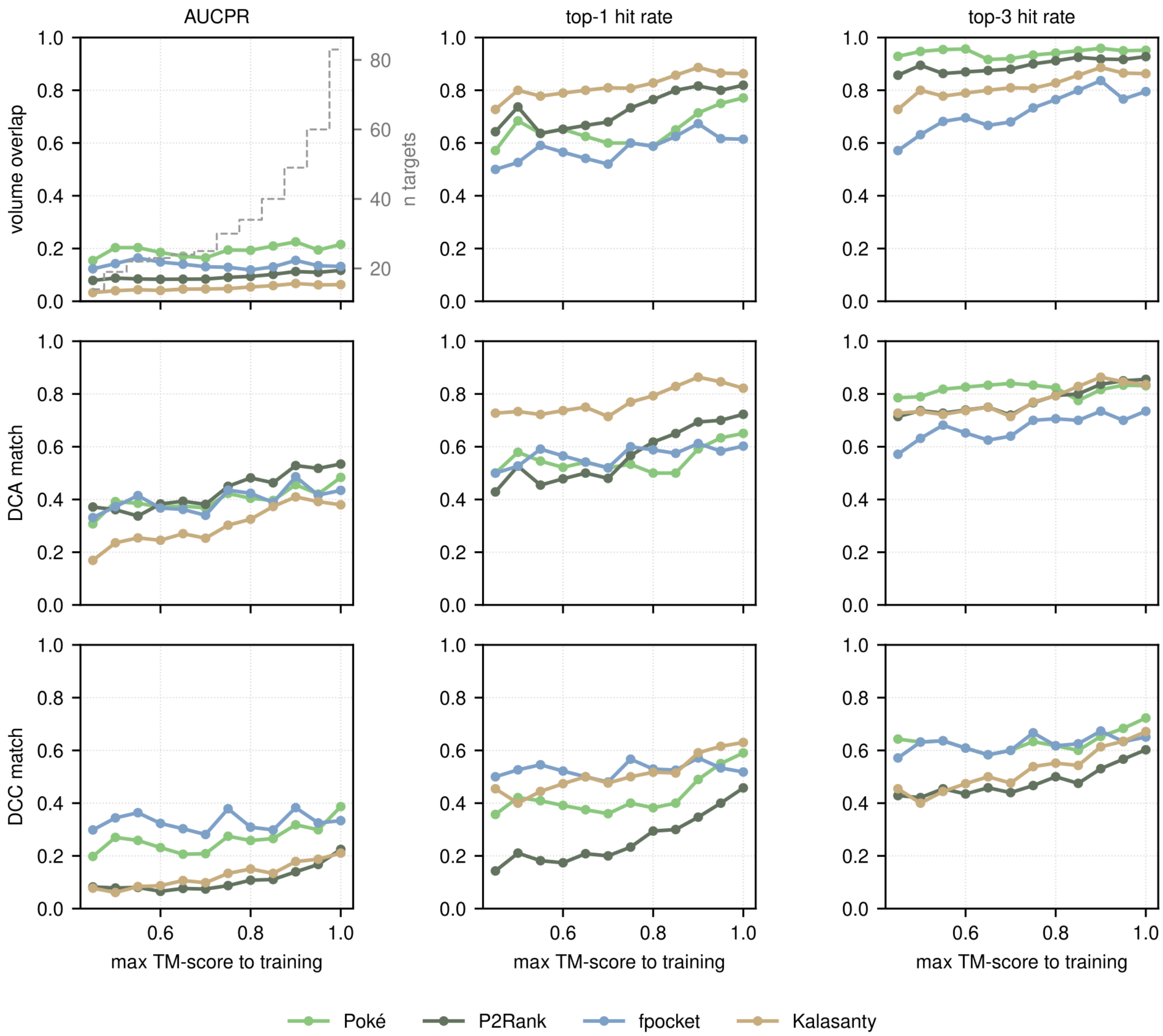

Figure S7. Pocket-detection performance as a function of structural similarity with the training partition. Detection accuracy on the Astex benchmark for Poké, P2Rank, fpocket and Kalasanty, stratified by each Astex target's structural similarity to the training set. Similarity is the maximum Foldseek TM-score (query-normalized) between a target's ligand-binding chain(s) and any binding chain in the fold-disjoint training set; the held-out structures, being excluded from training, are likewise excluded from the similarity reference. Each point is a cumulative subset comprising all targets with similarity ≤ the abscissa value: the leftmost points

therefore contain only the targets most dissimilar to training, the strongest test of generalization, while every subset toward the right additionally includes the more similar targets, so that the rightmost point (TM-score = 1.0) is the full Astex benchmark. This cumulative construction shows how performance on the complete dataset is approached as progressively more-similar targets are admitted, rather than partitioning the benchmark into separate slices. The gray dashed line in the top-left panel (right axis) gives the number of targets in each cumulative subset (subsets of fewer than 10 targets are not shown). Rows correspond to the three matching metrics (volume overlap, DCA, DCC); columns report the pooled area under the precision–recall curve (AUCPR, obtained by sweeping the pocket-score threshold over all pockets of all targets in the subset) and the per-target top-1 and top-3 hit rates. Specifically, the top-1 and top-3 metrics are defined as the fraction of targets for which at least one of the highest-1 or highest-3 scored pockets is a true positive (a pocket counts as a hit when within 4 Å of the ligand for DCA/DCC, or when it covers ≥ 25% of the ligand volume for volume overlap). Across all metrics, performance declines only gradually toward the low-similarity end and does not collapse for the targets most dissimilar to training, indicating that detection generalizes beyond near-duplicates of the training set rather than depending on close structural matches. Poké remains competitive across the full similarity range and retains its volume-overlap advantage even for the least similar targets.

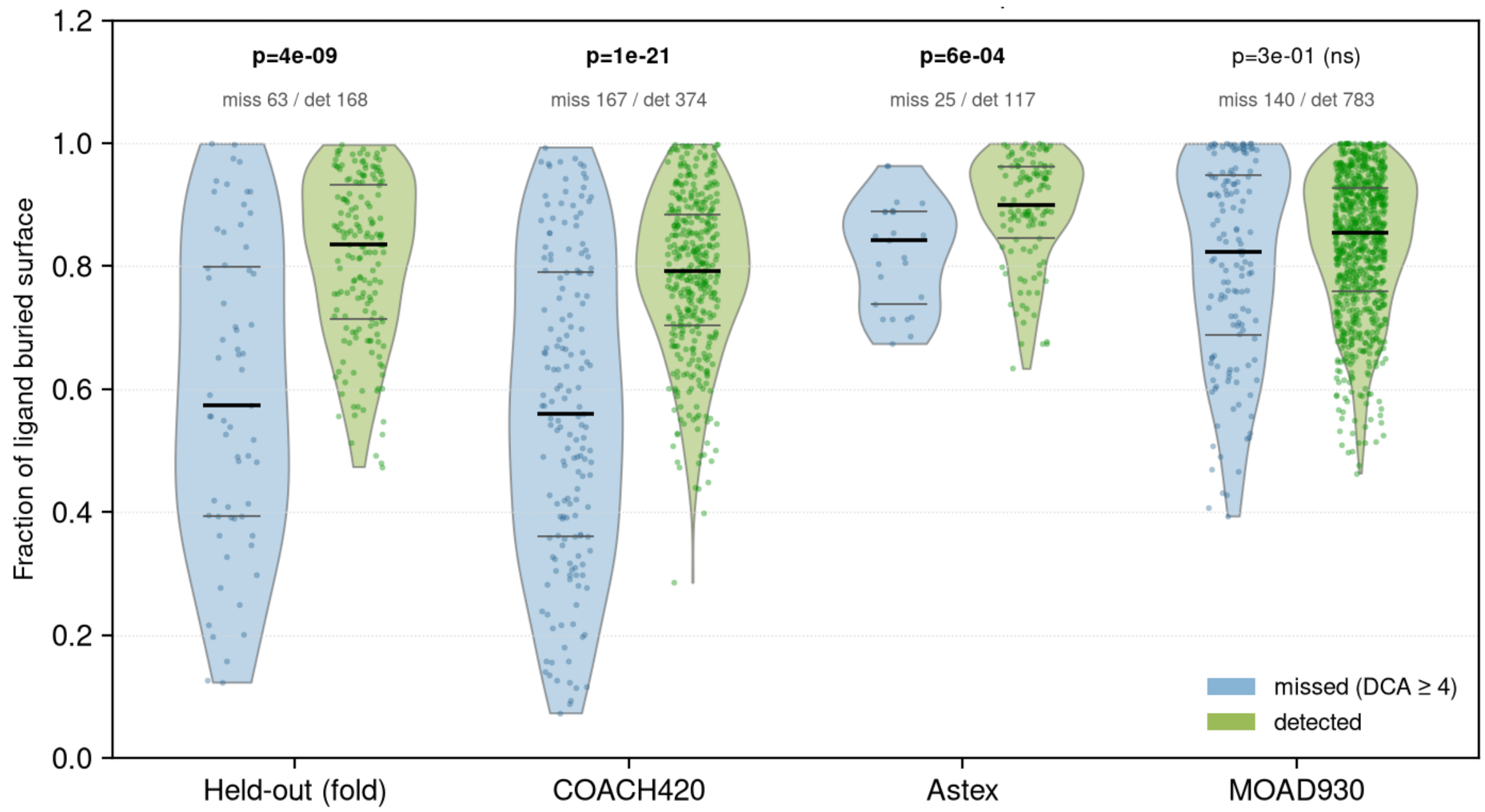

Figure S8. Detection failures are concentrated on solvent-exposed sites, across datasets. For each of four benchmark sets, the fraction of the bound ligand's solvent accessible surface (SASA) buried by the protein (1 – SASA_complex / SASA_free, computed with FreeSASA) is shown for the sites Poké misses (DCA ≥ 4 Å; blue) versus the sites it detects (green). Violins show the full distribution with the median (strong black line) and the 25th/75th percentiles; overlaid points are individual ligand copies (jittered horizontally for visibility). Missed sites are markedly less buried (more solvent-exposed) than detected ones on the fold-orthogonal held-out set (median buried fraction 0.57 vs 0.84; n = 63 vs 168), COACH420 (0.56 vs 0.79; n = 167 vs 374) and Astex (0.84 vs 0.90; n = 25 vs 117), but the effect is absent on the curated, deeply buried MOAD930 set (0.82 vs 0.85; n = 140 vs 783, not significant). p-values above each pair are Mann-Whitney U, tested per dataset ($p = 4 \times 10^{-9}$, 1×10^{-21}, 6×10^{-4} and 0.27

respectively). Pocket detection is model-independent (it is fixed by the scouting geometry, not the scoring model) so this comparison is unaffected by training-set membership.

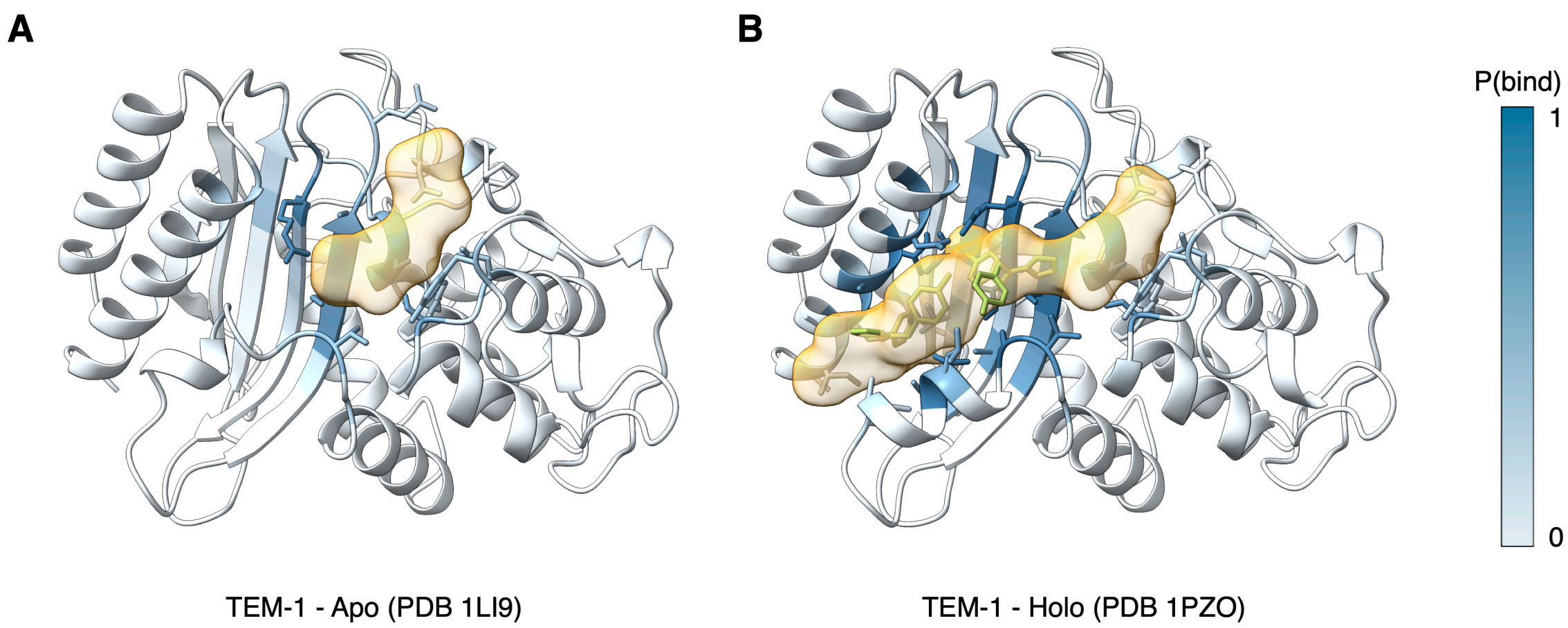

Figure S9. Poké and AF2BIND predictions on the apo and holo forms of TEM-1 β-lactamase. Poké and AF2BIND were run independently on the apo (A, PDB 1LI9) and holo (B, PDB 1PZO) structures of TEM-1 β-lactamase; the two structures are shown in the same orientation. In each panel the top-ranked pocket by Poké ligandability score is drawn as a transparent orange surface with its lining residues in sticks; the protein (both cartoon and sticks) is colored by the per-residue binding probability P(bind) predicted by AF2BIND; allosteric ligands are shown in green sticks. A) In the apo form the cryptic site is collapsed: Poké detects only the catalytic site (Poké score 6.3), and elevated P(bind) is likewise largely confined to that region. The AF2BIND recovery of the allosteric site is 0.36, in line with what is found in the original publication on apo PDB 1JWP. B) In the holo form, displacement of the α-helix spanning residues 213–229 opens the allosteric site, and Poké returns a single extended pocket encompassing both the allosteric and the catalytic sites (Poké score 6.6); AF2BIND assigns high

P(bind) across the same region. The calculated AF2BIND recovery of 0.68 reproduces the original publication.
